# Central Nervous System Infections Due to Aspergillus and Other Hyaline Molds

**DOI:** 10.3390/jof5030079

**Published:** 2019-08-30

**Authors:** Marisa H. Miceli

**Affiliations:** Division of Infectious Diseases, University of Michigan Health System, Ann Arbor, MI 48109, USA; mmiceli@med.umich.edu

**Keywords:** central nervous system, *Aspergillus*, hyaline molds

## Abstract

Central nervous system infections due to *Aspergillus* spp and other hyaline molds such as *Fusarium* and *Scedosporium* spp are rare but fatal conditions. Invasion of the central nervous system (CNS) tends to occur as a result of hematogenous dissemination among immunocompromised patients, and by local extension or direct inoculation secondary to trauma in immunocompetent hosts. Efforts should be directed to confirm the diagnosis by image-guided stereotactic brain biopsy when feasible. Non-culture methods could be useful to support the diagnosis, but they have not been validated to be performed in cerebral spinal fluid. Treatment of these infections is challenging given the variable susceptibility profile of these pathogens and the penetration of antifungal agents into the brain.

## 1. Introduction

Invasive mold infections are a cause of increased morbidity and mortality among immunosuppressed patients [1]. In particular, fungal infections of the central nervous system (CNS) usually have devastating consequences. This is due in part to their often-non-specific presentation, delayed diagnosis, and limited treatment options. Typically, neurosurgical procedures are not a therapeutic option due to the severity of patients’ underlying conditions and profound thrombocytopenia. This article presents an overview of the epidemiology, clinical presentation, diagnosis, and treatment of CNS infections due to *Aspergillus* and other hyaline molds.

## 2. *Aspergillus* Species

Invasive aspergillosis is the most common invasive mold infection, particularly among hematological cancer patients and peripheral stem cell transplant recipients [1]. *Aspergillus fumigatus* accounts for almost 90% of the infections in humans. Other non-*fumigatus Aspergillus* spp, including *A. flavus, A. terreus, A. niger, A.nidulans, A. ustus,* and *oryzae* are emerging as causes of infection. A high prevalence of non-*fumigatus Aspergillus* spp has been reported in tropical and subtropical geographic areas including Southeast Asia, the Middle East, and North America [2,3]. Most infections of the CNS in immunosuppressed hosts are due to *Aspergillus fumigatus*, while *A. flavus* is the predominant species among immunocompetent individuals [4]. However, infection due to other, less common species with intrinsic resistance to commonly used antifungal agents, may occur regardless of immune status.

### 2.1. Pathogenesis

Neuroaspergillosis may occur by hematogenous dissemination in the setting of disseminated infection, or by direct extension from the ear, paranasal sinuses, or mastoids in patients with localized invasive aspergillosis [4]. Hematogenous dissemination from invasive lung infection is more common among immunocompromised patients, while extension from sinusitis, mastoiditis, and direct penetration of the mold into the brain secondary to cranial trauma, injury, or neurosurgery are more commonly seen in immunocompetent hosts [4,5,6,7].

The intrinsic mechanisms by which *Aspergillus* spp penetrate the blood–brain barrier and produce damage of the central nervous system continue to be the subject of ongoing research. Studies have shown that *Aspergillus* spp produce mycotoxins (including aflatoxins and gliotoxins) that inhibit phagocytosis and reduce opsonization of the conidia during invasion [8,9,10,11]. Micotoxins have the ability to alter the integrity of the blood–brain barrier and damage and kill neurons, astrocytes and microglia [12]. In patients with profound immunosuppression, *Aspergillus* angioinvasion of the brain may result in cerebral infarction, hemorrhage, mycotic aneurysm, and meningitis [13]. Patients with a preserved immune system may be able to develop granulomas, brain abscesses and meningitis [14].

### 2.2. Clinical Presentation and Outcome

Clinical presentation of *Aspergillus* infection of the CNS is non-specific and varies depending on the extension of the infection. Indeed, *Aspergillus* invasion of the CNS may result in brain abscess, cerebritis, meningitis, cranial sinus thromboses, and ventriculitis. Therefore, patients may present with a variety of signs and symptoms, such as fevers, headaches, lethargy, altered mental status, seizures, abnormal gait, dizziness, or focal neurological findings [15]. Overall, the prognosis of patients with cerebral aspergillosis is poor. A recent study showed a median survival of 3.5 months after the diagnosis of CNS invasive mold infections, including aspergillosis, among patients with hematological malignancies [4]. In this study, mortality rate for mold infection as the primary cause of death was 33% (IFI CNS 29% vs. pulmonary IFI 46%, *p* = 0.32). Mortality from hematological malignancy disease with IFI as a contributory factor was 44%. One-year mortality rate was significantly higher among patients with CNS fungal infections compared to those with a pulmonary infection as a contributory factor (52% vs. 15%, *p* = 0.026).

### 2.3. Diagnosis

Brain imaging is essential in the diagnosis, but there are not specific radiological findings for the neuroaspergillosis. Computed tomography (CT) and magnetic resonance imaging (MRI) of the brain with contrast are critical to evaluate patients with suspected CNS involvement [16,17]. Intracranial dural enhancement may occur secondary to direct extension from sinuses with mucosal thickening and bone erosion. Ring-enhancing lesions consistent with abscesses are not uncommon. Patients with CNS aspergillosis secondary to direct invasion from the sinuses develop single lesions within the frontal or temporal lobe, while patients with hematogenous dissemination may present single or multiple lesions at the gray–white junction. Cerebral cortical and subcortical infarction, with or without hemorrhagic transformation and mycotic aneurysms, typically develop as result of angioinvasion [14,18].

The yield of image-guided stereotactic brain biopsy for microbiological and pathological diagnosis of focal lesions is high (80–90%), and it should be performed whenever possible [19]. Non-culture based methods for the diagnosis of invasive aspergillosis such as galactomannan (GM) and PCR have been only validated for their use in serum and bronchoalveolar lavage. 1,3-beta-di-glucan (BDG) has been validated and approved by the Food and Drug Administration for its use in serum only [20]. A positive test result of either of these biomarkers may support the diagnosis of CNS aspergillosis in the right setting. Off label testing of cerebral spinal fluid (CSF) with GM, BDG, or PCR could be helpful, particularly when performing brain biopsy is not an option. For instance, the detection of *Aspergillus* PCR, or a positive GM or BDG in the CSF of a patient suspected of cerebral aspergillosis, may support the diagnosis [21,22,23,24,25].

### 2.4. Treatment

Surgical excision of infected tissue such as paranasal sinuses, bone, and brain abscesses should be pursued when feasible [1]. A small retrospective study suggested that surgical resection of cerebral lesions in combination with antifungal therapy with voriconazole may improve survival [26].

For decades, the only therapeutic option for invasive aspergillosis was conventional amphotericin B (AmB). However, AmB compounds are large molecules and their CNS penetration is limited [27]. In addition, treatment is typically limited by renal and infusion related toxicities [28]. Liposomal formulations of AmB are associated with fewer toxicities and are usually better tolerated. Favorable responses of CNS aspergillosis in animal models and patients treated with lipid formulations of amphotericin B have been reported [29,30,31].

Of all available systemic antifungals, voriconazole achieves the widest distribution in the CSF, with CSF concentrations close to 50% of plasma concentration [27] (Table 1). Open-label studies of voriconazole vs. amphotericin B for the treatment of invasive aspergillosis showed a trend toward improvement of CNS aspergillosis among patients treated with voriconazole [26,32,33].

Current guidelines recommend the use of voriconazole (oral or IV) as a first line treatment of CNS aspergillosis [1,35]. Standard IV loading of voriconazole with 6 mg/kg every 12 h for two doses followed by 4 mg /kg Q12 h. Oral loading dose is 400 mg Q12 h × 2 doses, followed by 200 mg PO q 12 h. Duration of antifungal treatment is not clearly established and typically continues for several months, depending on the clinical and radiological response and ongoing immunosuppression.

Voriconazole toxicities are more common among patients with elevated drug concentrations in serum [36]. For instance, hepatotoxicity and peripheral and central neurologic symptoms and visual hallucinations are typically observed in patients with higher drug concentration levels [37]. Therapeutic drug monitoring is useful to optimize the efficacy and safety of voriconazole, particularly among patients receiving drugs that may affect voriconazole serum concentrations such as antiseizure medications (phenytoin, phenobarbital, others) [1,35].

Similar to AmB, itraconazole, and posaconazole are large molecules (>700-dalton) and they exhibit limited penetration to the CNS (Table 1), but have been successfully used in patients who were refractory to, or intolerant of, conventional therapy with voriconazole [38,39,40,41].

Echinocandins are active against *Aspergillus* spp, but their use (alone or in combination) should be limited to salvage therapy. Biodistribution of echinocandins to the eye and uninfected CSF is low. Their use for CNS aspergillosis is not recommended. However, successful cases treated with caspofungin and micafungin have been reported [42,43].

European guidelines include isavuconazole as an option for the treatment of invasive aspergillosis, including cerebral aspergillosis [35]. Animal studies have demonstrated efficient penetration and homogeneous distribution patterns of isavuconazole in the infected brain [34]. In addition, there have been some reports of patients with CNS aspergillosis successfully treated with this drug [44,45].

In addition to antifungal therapy, immunosuppression should be reduced or reversed whenever possible [1]. Currently, there is no data to support the use of combination antifungal therapy for CNS aspergillosis, although some animal studies and case reports have shown favorable responses. Similarly, the use of corticosteroids and intrathecal antifungal therapy may be detrimental, and is not recommended [1,46].

**Figure 1:**
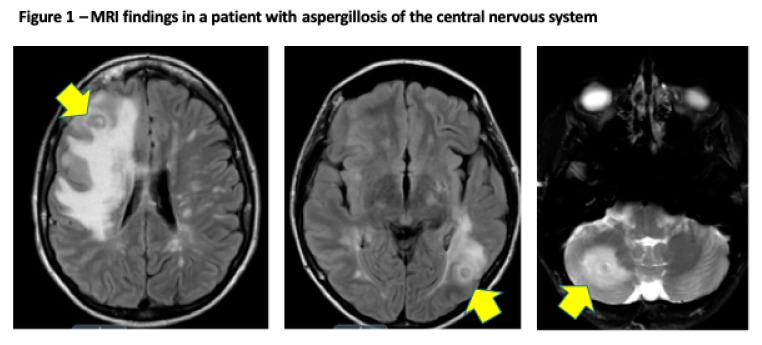
Proven cerebral aspergillosis in a 30-year old female diagnosed within 45 days after orthotopic liver transplantation. MR of the brain shows hyperintense T2 and FLAIR signal throughout the cerebral white matter, and deep gray nuclei structures with discrete ring enhancing lesions and surrounding edema seen in the left occipital lobe and right cerebellum (arrows).

Figure 1 illustrates a case of proven cerebral aspergillosis in a liver transplant recipient, diagnosed on the bases of clinical presentation, MRI brain, and brain biopsy. Patient was treated with voriconazole and reduction of immunosuppression (mycophenolate). Antifungal therapy was continued beyond 12 months from diagnosis, with significant reduction of the size of the brain lesions.

## 3. Other Hyaline Molds

### 3.1. Fusarium Species

In recent years, there has been an increase in the number of invasive infections due to *Fusarium* spp in patients with underlying immunosuppression. *Fusarium* spp are the second most frequent cause of invasive mold infection in immunocompromised patients after Aspergillus spp [47,48,49]. *Fusarium solani* is the most frequent species causing invasive disease in patients with hematological malignancies, stem cell transplant recipients, and prolonged neutropenia [50,51,52]. Similar to *Aspergillus* spp, *Fusarium* spp has the ability to produce mycotoxins (trichothecenes and fumonisins); the main role of these virulence factors is suppression of humoral and cellular immunity. The most common mycotoxin produced by *Fusarium* spp is fumonisin B1, which has been associated with cerebral invasion, causing neural axon degeneration, and abnormal mitochondrial function [53]. Cerebral fusariosis is typically diagnosed in the setting of disseminated infection [54]. Patients may develop single or multiple brain abscesses, meningitis, endophthalmitis, chorioretinitis, cutaneous nodules, and fungemia [55]. The most common species include *F. solani*, *F. oxysporum,* and *F. moliniforme* complexes [47]. Similar to other invasive mold infections, definitive diagnosis is made by direct pathological exam and identification of the organism invading tissue (brain biopsy, CSF, vitreal fluid). *Fusarium* spp can cause fungemia and therefore, the diagnosis can sometimes be made on the basis of positive blood cultures [52]. Non-cultural methods such as GM and BDG in serum, BAL, and CSF are non-specific and could be positive in patients with invasive fusariosis [20].

Treatment of invasive fusariosis is challenging given the severity of this infection, variable antifungal susceptibility, and profound immunosuppression of the host. Typically, these patients start a combination of lipid formulation AmB and voriconazole, pending susceptibilities.

### 3.2. Scedosporium Species

Most common *Scedosporium* species causing infection in humans include *S. apoiospermum* and *S. proliferans* (now *Lomentospora prolificans*). Most CNS infections due to *S. apiospermum* occur in immunocompetent patients, typically associated with near drowning, motor vehicle accidents, or direct inoculation (neurosurgery, CSF drainage devices, shunts, etc.) [56,57,58]. Cerebral scedosporiosis due to *L. proliferans* has been described in immunocompromised patients, is typically due to rapid hematogenous dissemination, and is invariably fatal [55,59]. Most cases in immunocompetent hosts are due to direct inoculation (trauma or surgery). Diagnosis is made by direct observation of the mold in histology or recovery in sterile cultures (brain, CSF). Of note, *L. proliferans* may be recovered in blood cultures, which may facilitate the diagnosis. Non-cultural tests are non-specific and could be positive in patients with scedosporidiosis, supporting the diagnosis. Antifungal susceptibilities of *Scedosporium apiospermum* are variable. Voriconazole should be used as initial therapy while antifungal susceptibilities are pending. Although mortality is high in patients with disseminated infection, a voriconazole minimum inhibitory concentration (MIC) <2 μg/mL has been associated with favorable outcomes [60]. *L. proliferans*, on the other hand, is resistant to all antifungal classes currently available. The combination of two antifungal agents has become a therapeutic alternative against *S. apiospermum* and *L. proliferans* [61,62,63,64]. In vitro studies have shown synergistic effects of voriconazole in combination with amphotericin B or echinocandins, against both *S. apiospermum* and *L prolificans*. An in vitro synergistic effect against *L. prolificans* has also been reported with terbinafine in combination with voriconazole, miconazole, or itraconazole [65,66]. Despite this promising in vitro data, the treatment of these infections is challenging, and outcomes are variable. Adjunctive surgical debridement and reversal of immunosuppression should be pursued when possible.

## Figures and Tables

**Table 1:** Antifungal agents and central nervous system penetration.

Antifungal Agent	Molecular Mass	Protein Binding	CSF Concentration *	Comment
Voriconazole	349 Daltons	58%	~50%	Small moderately lipophilic molecule
Posaconazole	708 Daltons	>98%	Very low to undetectable	Largest lipophilic compound, limited data
Itraconazole	705 Daltons	99.8%	<10%	Lipophilic compound
Isavuconazole	437 Daltons	>99%	Low	Water soluble. High concentration in eyes and brain
Amphotericin B and lipid formulations	924 Daltons	90%	Poor in adults; 40–90% in neonates Limited data with lipid formulations	Large molecule with a hydrophilic polydroxyl chain and a lipophilic polyene hydrocarbon chain, poorly soluble in water
Echinocandins	1140–1292 Daltons	97–99%	Negligible	Brain tissue concentration may increase with dose escalation and prolonged exposure

* percentage of plasma concentration, data from animal and human studies [27,34].
